# Quantification of Aluminum Gallium Arsenide (AlGaAs) Wafer Plasma Using Calibration-Free Laser-Induced Breakdown Spectroscopy (CF-LIBS)

**DOI:** 10.3390/molecules27123754

**Published:** 2022-06-10

**Authors:** Tahani A. Alrebdi, Amir Fayyaz, Haroon Asghar, Asif Zaman, Mamoon Asghar, Fatemah H. Alkallas, Atif Hussain, Javed Iqbal, Wilayat Khan

**Affiliations:** 1 Department of Physics, College of Science, Princess Nourah bint Abdulrahman University, P.O. Box 84428, Riyadh 11671, Saudi Arabia; taalrebdi@pnu.edu.sa (T.A.A.); fhalkallas@pnu.edu.sa (F.H.A.); 2 National Centre for Physics, Quaid-i-Azam University Campus, Islamabad 45320, Pakistan; amirfayyaz@phys.qau.edu.pk; 3 Department of Physics, Islamia College, Peshawar 25000, Pakistan; asifzaman@icp.edu.pk; 4 Department of Physics, University of Gujrat, Hafiz Hayat Campus, Gujrat 50700, Pakistan; mamoonasghar319@gmail.com; 5 Department of Physics, The University of Lahore, Gujrat Campus, Gujrat 50700, Pakistan; atif.hussain@phys.uol.edu.pk; 6 Department of Physics, Azad Jammu and Kashmir University, Muzaffarabad 13100, Pakistan; javedkiqbal@gmail.com; 7 Department of Physics, Bacha Khan University, Charsadda 24420, Pakistan; walayat76@gmail.com

**Keywords:** CF-LIBS, AlGaAs, quantitative analysis, plasma parameters, compositional analysis

## Abstract

In this work, we report the results of the compositional analysis of an aluminum gallium arsenide (AlGaAs) sample using the calibration-free laser-induced breakdown spectroscopy (CF-LIBS) technique. The AlGaAs sample was doped with three various concentrations of gallium (Ga), arsenic (As), and aluminum (Al), as reported by the manufacturer, and the CF-LIBS technique was employed to identify the doping concentration. A pulsed Q-switched Nd: YAG laser capable of delivering 200 and 400 mJ energy at 532 and 1064 nm, respectively, was focused on the target sample for ablation, and the resulting emission spectra were captured using a LIBS 2000+ spectrometer covering the spectral range from 200 to 720 nm. The emission spectra of the AlGaAs sample yielded spectral lines of Ga, As, and Al. These lines were further used to calculate the plasma parameters, including electron temperature and electron number density. The Boltzmann plot method was used to calculate the electron temperature, and the average electron temperature was found to be 5744 ± 500 K. Furthermore, the electron number density was calculated from the Stark-broadened line profile method, and the average number density was calculated to be 6.5 × 10^17^ cm^−3^. It is further observed that the plasma parameters including electron temperature and electron number density have an increasing trend with laser irradiance and a decreasing trend along the plume length up to 2 mm. Finally, the elemental concentrations in terms of weight percentage using the CF-LIBS method were calculated to be Ga: 94%, Al: 4.77% and As: 1.23% for sample-1; Ga: 95.63%, Al: 1.15% and As: 3.22% for sample-2; and Ga: 97.32%, Al: 0.69% and As: 1.99% for sample-3. The certified concentrations were Ga: 95%, Al: 3% and As: 2% for sample-1; Ga: 96.05%, Al: 1% and As: 2.95% for sample-2; and Ga: 97.32%, Al: 0.69% and As: 1.99% for sample-3. The concentrations measured by CF-LIBS showed good agreement with the certified values reported by the manufacturer. These findings suggest that the CF-LIBS technique opens up an avenue for the industrial application of LIBS, where quantitative/qualitative analysis of the material is highly desirable.

## 1. Introduction

During the last few decades, various techniques, including atomic absorption spectroscopy (AAS) [1,2], inductively coupled plasma optical emission spectroscopy (ICP-OES) [3], inductively coupled plasma mass spectroscopy (ICP-MS) [4], and X-ray fluorescence [5], have been utilized for the quantitative analysis of the material. Of all these techniques, laser-induced breakdown spectroscopy (LIBS) has been proven to be an emerging, simple, well-established, robust, reliable, and cost-effective technique for elemental analysis [6,7,8,9,10,11]. The LIBS technique has many potential applications in the field of geology [12,13], industry [14], medical [15], and environmental studies [16]. In LIBS, an emission spectrum of the target material is recorded by focusing a laser beam on the surface of a target sample, which generates plasma on the surface in the form of free electrons, ions, and neutral atoms [17,18]. The plasma contains all the compositional elements present in the sample. Due to the hot nature of plasma, excitation and de-excitation processes happen within the plasma, which yields spectral lines that provide qualitative and quantitative information about the sample. In recent years, the LIBS technique has been used for the analysis of nanomaterials [19], thin-film analysis [20,21,22], and the identification of elements in semiconductor materials [23,24,25]. AlGaAs is an important crystalline solid used as a semiconductor material, which has potential photo-optic practical applications. In addition, AlGaAs is used as a barrier material in GaAs-based diode lasers. The performance and efficacy of AlGaAs-based diode lasers have a strong dependence on the precise percentage of doping of semiconductor materials, including Al, Ga and As. Hence, it is crucial to have precise knowledge of the required concentration of elements present in the semiconductor materials to acquire the desired efficiency and better applications [26].

Here, we present the qualitative and quantitative analysis of an AlGaAs-based semiconductor material using the CF-LIBS technique, and the results were compared with results reported by the manufacturer. Three different samples with various concentrations of Al, Ga, and As were chosen, and a compositional analysis was performed using the CF-LIBS technique. Elements present in the sample, including Ga, As and Al, were identified from the spectral lines present in the recorded emission spectra of the AlGaAs sample. The electron temperatures of Ga, As, and Al were calculated using the Boltzmann plot method, while the number density was calculated using the Stark-broadened line profile method. The average electron temperature and electron number density were used for quantitative analysis of the target sample. The elemental concentration, in terms of weight percentage, was calculated using the CF-LIBS technique and the results were compared with those reported by the manufacturer. The measured concentration by CF-LIBS showed good agreement with the certified values reported by the manufacturer.

## 2. Experimental Setup

**Figure 1:**
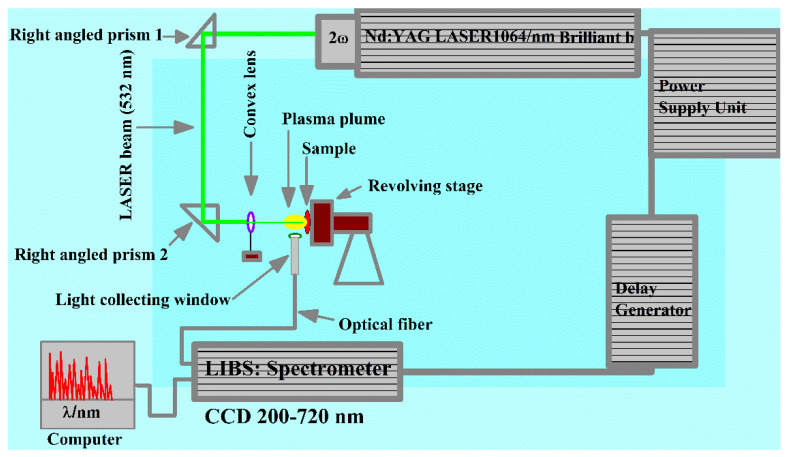
Experimental LIBS setup used to study the laser-generated plasma of the AlGaAs sample.

The LIBS experimental setup used to investigate the compositional analysis of the sample has been explained elsewhere [7,8,9,10,11]. A schematic diagram of the LIBS arrangement used for the study of the AlGaAs sample is shown in Figure 1. In brief, the LIBS system contains a pulsed Nd: YAG laser (Brilliant-B, Quantel, Lannion, France), which emits at 532 nm wavelength, operates at 10 Hz repetition rate with 5 ns pulse duration, and is capable of delivering about 200 mJ of pulse energy. The laser beam, having a pulse energy of about 118 mJ, was focused onto the surface of the target sample, which was positioned in air at atmospheric pressure using a quartz lens of 10 cm focal length. The AlGaAs sample was placed on a rotating stage to enable a clean surface for every laser shot. The optical emission was recorded using an optical fiber attached to a spectrometer equipped with charged coupled devices (CCDs) that range from 200 nm to 720 nm wavelengths. Ten laser shots were used to clean the sample surface. The optical emission spectra were accomplished at an average of 20 laser shots at distinct spots on the surface of the sample. The averaged optical emission spectrum was then utilized to acquire the chemical composition of the AlGaAs sample, which takes into account the sample inhomogeneity and lowers the statistical errors.

## 3. Material

Gallium arsenide (GaAs) belongs to the III–V group of semiconductor elements. GaAs has many unique qualities, such as low electronic effective mass, high electron mobility, and high-saturation drift velocity. GaAs is used in various devices, such as optoelectronics and microelectronics. Therefore, for the past couple of years, excellent studies have been conducted to investigate the chemical and physical properties of its nanostructure [27]. In the present work, to study the optical emission spectrum of AlGaAs, we used three standard certified AlGaAs samples with varying concentrations of Al, Ga and As. The details of the AlGaAs-certified samples are presented in Table 1.

## 4. Results and Discussions

### 4.1. LIBS Emission Studies

**Figure 2:**
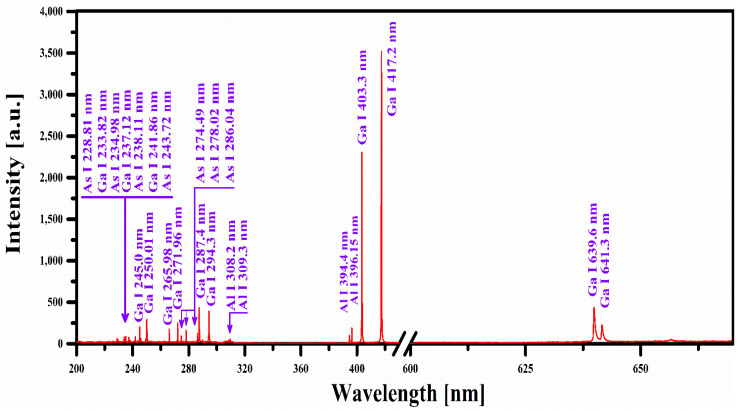
Optical emission spectrum of the AlGaAs sample covering the wavelength range from 200 nm to 670 nm.

We recorded the emission spectrum of the AlGaAs samples under identical LIBS conditions; however, a comprehensive study has been conducted for sample-1. In Figure 2, we present the time-integrated emission spectrum of AlGaAs for sample-1, and the characteristic emission lines of Ga (I), As (I), and Al (I) are marked. In this spectral region, the dominant emission lines are attached to singly ionized gallium (Ga I) at 403.30 nm due to 4s^2^5s ^2^S_1/2_→3d^10^4s^2^4p ^2^P_1/2_ transition, and 417.20 nm due to 4s^2^5s ^2^S_1/2_→3d^10^4s^2^4p ^2^P_3/2_ transition, followed by singly ionized arsenide (As I) and singly ionized aluminum (Al I) lines. The identification of the spectral lines belonging to various elements was accomplished using the National Institute of Standard and Technology (NIST) database [28]. The detected major and minor emission lines of sample-1, along with their wavelengths, transition configuration, transition probabilities, and upper level energies, are presented in Table 2. These optical emission lines are used to estimate the concentrations of the elements Ga, As, and Al. For the quantitative analysis of the AlGaAs samples, plasma parameters, such as plasma temperature and electron number density, were calculated by assuming that the plasma is optically thin and in local thermodynamic equilibrium (LTE).

### 4.2. Plasma Temperature (T_e_)

**Figure 3:**
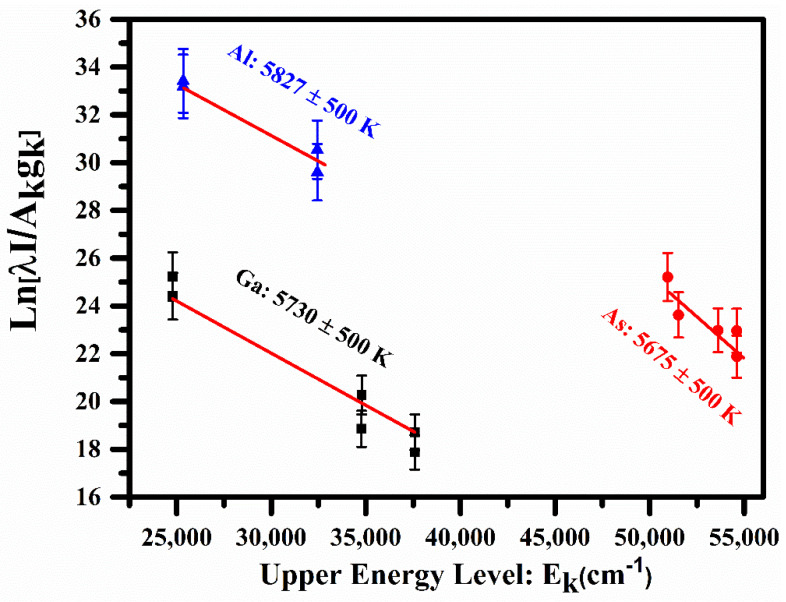
Typical Boltzmann plots for sample-1 using several neutral emission lines of Ga, As, and Al.

To investigate the laser-produced plasma of the AlGaAs sample, certain conditions should be satisfied, including optically thin plasma and local thermodynamic equilibrium (LTE) conditions. To validate the above assumptions, it is essential to evaluate the plasma temperature (*T_e_*) and electron density (*N_e_*). Several methods and techniques for determining the plasma temperature and number density have been utilized in previous LIBS studies [18,29,30]. However, in the present work, we used the Boltzmann plot method for measuring the plasma temperature. By assuming that the plasma population is obeying the Boltzmann distribution, we used the following Boltzmann equation to construct the Boltzmann plots [31,32]:

(1) $$\ln\left(\frac{I_{ij}\lambda_{ij}}{hcA_{ij}g_{i}}\right)=-\frac{E_{i}}{kT_{e}}+ln\left(\frac{N_{e}}{P\left(T_{e}\right)}\right)$$

where *I_ij_* is the spectral line intensity of the transition *j*→*i*, λ is the transition wavelength, *h* is the Planks constant, *A* is the transition probability, $g_{i}$ is the statistical weight of the upper level, *c* is the velocity of light, $E_{i}$ is the energy of the upper level, k is the Boltzmann constant, *T_e_* is the excitation temperature, $N_{e}$ is the total number density, and $P\left(T\right)$ is the partition function. To draw the Boltzmann plot, we have selected the optically thin emission lines of Ga I, As I, and Al I that are free from self-absorption and also follow the local thermodynamical equilibrium (LTE). The constructed Boltzmann plots for Ga I, As I, and Al I are presented in Figure 3, displaying excellent linearity (R^2^~0.999). The plasma temperatures have been obtained from the slopes (1/*kT_e_*) of the linear fit. The calculated plasma temperatures for Ga, As, and Al are (5730 ± 500 K), (5675 ± 500 K), and (5827 ± 500 K), respectively. The selected emission lines and their atomic parameters were taken from the NIST database and are listed in Table 2. The errors in the calculated plasma temperatures mainly come from the uncertainties present in the reported transition probabilities and the measurement of the line intensities. For the quantitative analysis, we have used a mean value of the plasma temperature, (5744 ± 500 K).

To validate the conditions of being optically thin and free from self-absorption, we used the intensity ratio method in the present study [18,30,33]. For instance, upper-level energies were used to lower the temperature dependency. The intensity ratios of the experimentally observed spectral lines and values calculated from the atomic parameters (NIST database 2022 [28]) are quite compatible (error <10%). Thus, the plasma satisfies the condition of being optically thin.

### 4.3. Plasma Electron Number Density (N_e_)

**Figure 4:**
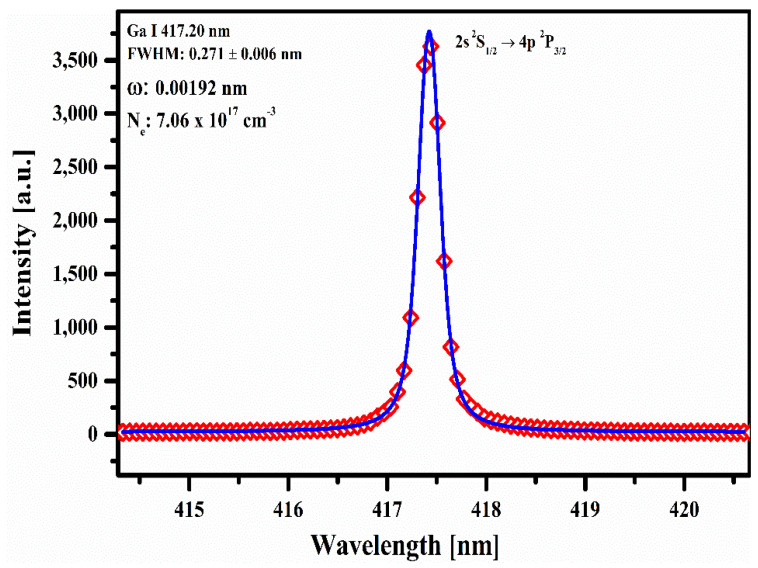
A typical Stark-broadened line profile of Ga I at 417.20 nm along with the Voigt fitting profile.

The electron number density (*N_e_*) was calculated from the full width at half maximum (FWHM) of the Stark-broadened line profile of the neutral gallium (Ga I) at 417.20 nm [34,35].

(2) $$N_{e}\left({\mathrm{cm}}^{-3}\right)=\frac{\Delta \lambda_{FWHM}^{S}}{2\omega_{s}\left(\lambda ,T_{e}\right)}\times N_{r}$$

where $\omega_{s}$ is the Stark-broadened parameter (0.00192 nm) for this Ga I emission line, and *N_r_* is the reference electron number density, which is $10^{16}{\mathrm{cm}}^{-3}$ for the neutral line. A Voigt fitting profile (Cauchy–Lorentz distribution and a Gaussian distribution) of the Ga I emission line at 417.20 nm, which takes into account the instrumental resolution $\left(\sim 0.06\;\mathrm{nm}\right)$ and the Doppler width $\left(\sim 0.0036\;\mathrm{nm}\right)$, is shown in Figure 4. The electron number density is determined as: $\left(7.06\pm 0.1\right)\times 10^{17}{\mathrm{cm}}^{-3}$.

### 4.4. Local Thermodynamical Equilibrium (LTE)

McWhirter’s criterion is used to check the lower limit of the electron number density for the plasma to be in local thermodynamic equilibrium (LTE) [31,36,37].

(3) $$N_{e}\left({\mathrm{cm}}^{-3}\right)\geq 1.6\times 10^{12}{\left(T_{\left(K\right)}\right)}^{1/2}{\left(\Delta E_{\left(eV\right)}\right)}^{3}$$

where $\Delta E\left(eV\right)$ is the highest energy transition between the upper and lower levels, and $T_{\left(k\right)}$ is the plasma excitation temperature. In this work, $\Delta E\left(eV\right)=2.97\;eV$ for Ga I and $T_{\left(k\right)}=5744\;K$. The calculated electron number density using this relation is at the order of ~$10^{15}{\mathrm{cm}}^{-3}$. This value of the number density is much lower than that of ($7.06\pm 0.1)\times 10^{17}{\mathrm{cm}}^{-3}$ determined from the Stark-broadened line profile of Ga I line at 417.20 nm. Therefore, it can be concluded that the plasma is satisfying the LTE condition.

### 4.5. Laser Irradiance and Spatial Dependence on the Plasma Parameters

**Figure 5:**
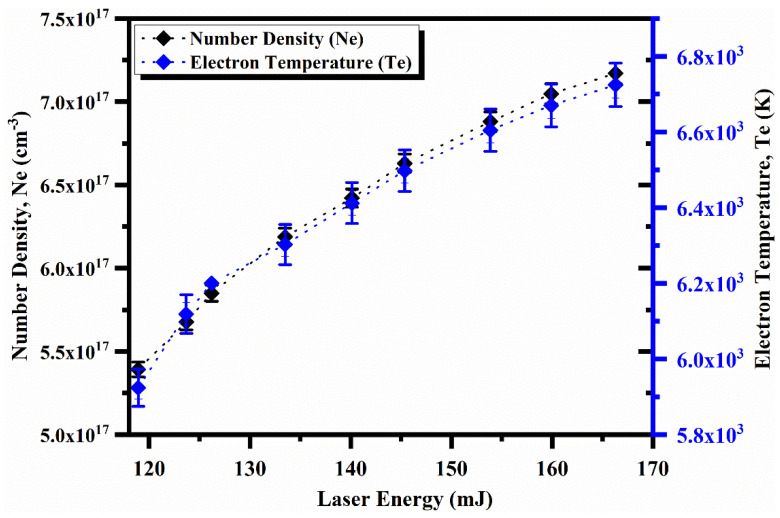
Variation in the electron temperature and the electron number density for Ga as a function of laser irradiance.

In the following section, the absorption of laser energy into the plasma has been investigated, as it depends on the plasma’s nature and the laser irradiance. Hence, a comprehensive study of spectroscopic parameters, including electron temperature and electron number density, is highly desirable. The electron number density and electron temperature as a function of laser energy are shown in Figure 5. The measured data demonstrate that the electron temperature and electron number density vary from 5800 K to 6700 K and 5.25 × 10^17^ cm^−3^ to 7.25 × 10^17^ cm^−3^, respectively. The increase in electron temperature and electron number density happens due to high laser irradiance, which generates a higher number of free electrons and hot plasma. Furthermore, the increasing trend in electron temperature and electron number density occurs because plasma formation and laser absorption take place simultaneously [38].

**Figure 6:**
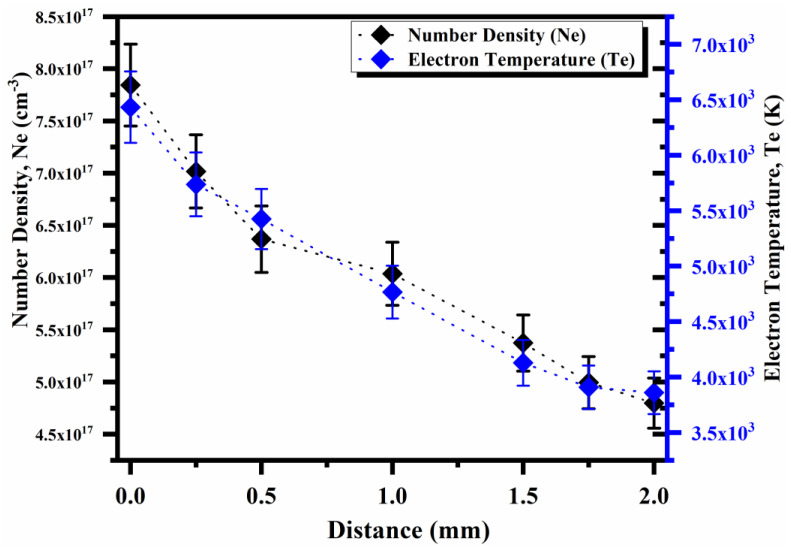
Variation in the electron temperature and the electron number density for Ga as a function of distance.

We further studied the spatial behavior of the electron temperature and electron number density when the distance along the direction of propagation of the plasma plume is varied from the target surface. The maximum electron temperature was noticed to be 6500 K and the minimum temperature was found to be 4000 K at distances of 0 and 2 mm, respectively. The electron temperature and electron number density as a function of distance from the target surface are shown in Figure 6. It is pertinent to mention here that the temperature is high near the target surface due to the absorption of laser radiations by electrons through the inverse bremsstrahlung absorption process, thus resulting in high average kinetic energies of particles and, hence, a high temperature. However, a decrease in the electron temperature with decreasing distance occurs because thermal energy is converted into kinetic energy when the plasma is expanding, so the plasma cools down along its direction of expansion.

## 5. Chemical Composition by CF-LIBS

After calculating the plasma parameters, the chemical composition was estimated using parameters such as electron number density and plasma temperature. We used the calibration-free (CF–LIBS) technique to estimate the chemical composition of the three AlGaAs samples, which is discussed in detail elsewhere [39,40,41]. The atomic concentration of the neutral atoms is determined using the well-known Boltzmann equation [7,9,10,11,35,39].

(4) $$FW^{\gamma }=I_{ki}\frac{P^{\gamma }\left(T\right)}{A_{ki}g_{k}}\exp\left[\frac{E_{k}}{k_{B}T}\right]$$

The factor *F* is constant for constant efficiency of the spectral system, which can be determined by normalizing the concentrations of the elements present in the sample. $W^{\gamma }$ is the concentration of the neutral atom, $P^{\gamma }\left(T\right)$ is the partition function that is temperature dependent, $I_{ki}$ is the integrated transition line intensity, $g_{k}$ is the statistical weight, $A_{ki}(s^{-1}$) is the transition probability, *E_k_* (eV) is the energy of the upper level, T is the excitation temperature (eV), and *k_B_* is the Boltzmann constant. All the atomic factors used for the analysis were taken from the NIST database [28]. The concentrations ($W^{\gamma }$) of the neutral atoms in the sample are calculated using Equation (4). To calculate the concentration of the ionized species, the Saha–Boltzmann equation was used [29,30,33,35]:

(5) $$N_{e}\frac{W^{\alpha ,\gamma +1}}{W^{\alpha ,\gamma }}=6.04\times 10^{21}\sqrt[{3}]{T_{eV}}\;\frac{U_{\alpha ,\gamma +1}}{U_{\alpha ,\gamma }}\;exp\left[-\frac{E_{\alpha ,\gamma }}{k_{B}T}\right]$$

Here, $E_{\alpha ,\gamma }\;\left(eV\right)$ is the ionization energy of the element *α*, $N_{e}\;\left({\mathrm{cm}}^{-3}\right)$ is the electron number density, $W^{\alpha ,\;\gamma +1}$ is the concentration of the *γ*+1 charge state, and $U_{\alpha ,\gamma +1}$ and $U_{\alpha ,\gamma }$ are the partition functions of the upper charge state (*γ*+1) and lower charge state (*γ*), respectively. The contribution of any element present in the sample is the sum of the neutral and ionic contributions [35,39].

(6) $$W^{\alpha }=W^{\alpha ,\gamma }+W^{\alpha ,\gamma +1}$$

(7) $$W^{\alpha }\left(\%\right)=\frac{W^{\alpha }}{\sum W^{\alpha }}\times 100$$

The concentration (%) of each ingredient in the sample is calculated using Equation (7).

**Figure 7:**
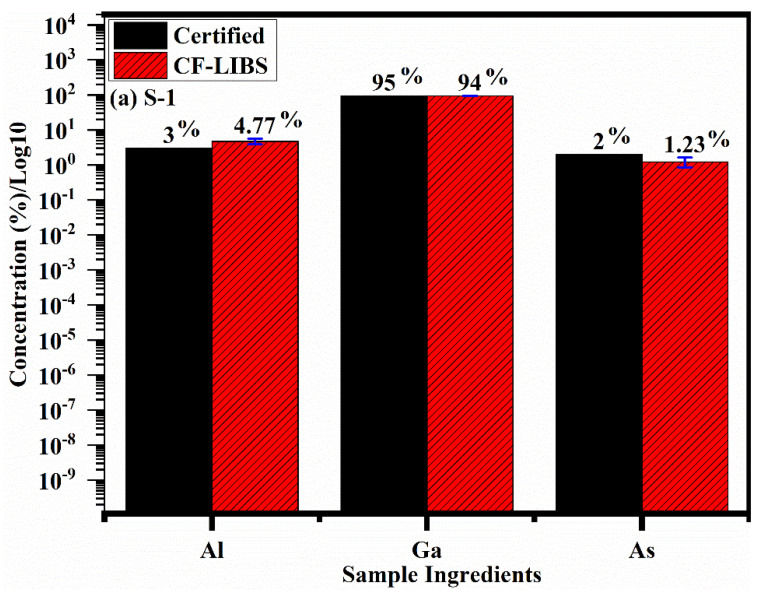

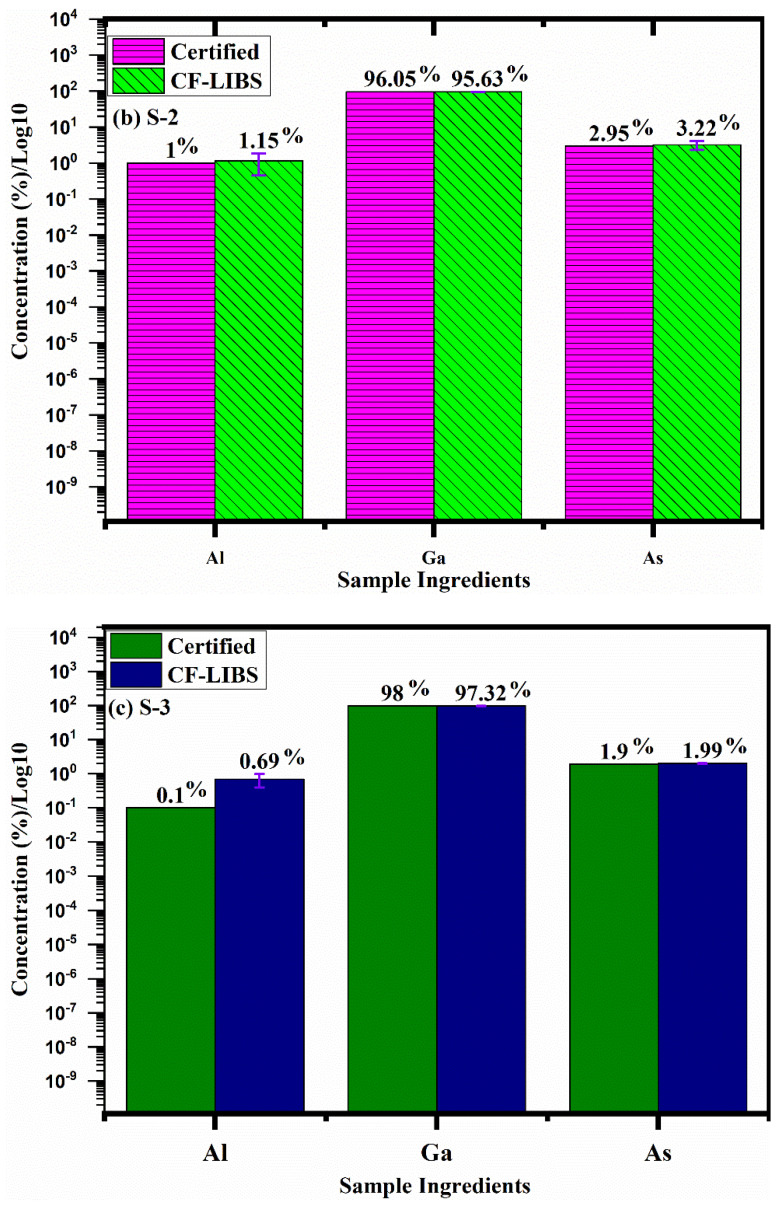
A histogram across elements versus concentration with varying wt.% of Al, Ga and As for both certified and CF–LIBS. (**a**–**c**) bar chart shows the comparison of concentration calculated by the CF-LIBS technique with that of the certified in the three samples specifically S–1, S–2, and S–3.

The calculated concentrations (%) of the three GaAs samples (S-1, S-2, S-3) using the CF-LIBS technique are presented in Figure 7a–c. These results demonstrate that the elemental concentration using the CF-LIBS method and their concentration in terms of weight percentage were calculated to be Ga: 94%, Al: 4.77% and As: 1.23% for sample-1; Ga: 95.63%, Al: 1.15% and As: 3.22% for sample-2; and Ga: 97.32%, Al: 0.69% and As: 1.99% for sample-3. However, the certified concentrations were Ga: 95%, Al: 3% and As: 2% for sample-1; Ga: 96.05%, Al: 1% and As: 2.95% for sample-2; and Ga: 97.32%, Al: 0.69% and As: 1.9% for sample-3. The measured concentration by CF-LIBS showed good agreement with the certified values reported by the manufacturer. The error bar (violet color) in the graph shows standard deviation in the concentration of the samples calculated using CF-LIBS and with that of the certified wt.%. This comparison shows the effectiveness and robustness of the CF-LIBS technique for the elemental analysis of the sample.

## 6. Conclusions

In summary, we present the qualitative and quantitative analysis of an AlGaAs wafer using the CF-LIBS technique. An AlGaAs sample was doped with three chosen concentrations of Ga, Al, and As, as reported by the manufacturer, and the CF-LIBS technique was used to verify the doping concentration. The recorded emission spectra of the AlGaAs sample yielded spectral lines of Ga, As and Al. The Boltzmann plot method was used to calculate the electron temperature, and the electron number density was calculated from the Stark-broadened line profile method. The average electron temperature and electron number density were used for the compositional analysis. Besides the variation in electron temperature and electron number density as a function of laser irradiance and distance was also discussed. Finally, the elemental concentration was calculated using the CF-LIBS method and their concentrations in terms of weight percentages were calculated to be Ga: 94%, Al: 4.77% and As: 1.23% for sample-1; Ga: 95.63%, Al: 1.15% and As: 3.22% for sample-2; and Ga: 97.32%, Al: 0.69% and As: 1.99% for sample-3. However, the certified concentrations were Ga: 95%, Al: 3% and As: 2% for sample-1; Ga: 96.05%, Al: 1% and As: 2.95% for sample-2; and Ga: 97.32%, Al: 0.69% and As: 1.99% for sample-3. The measured concentration by CF-LIBS showed good agreement with the certified values reported by the manufacturer. These results indicate that CF-LIBS is a compatible and potential technique to estimate the composition of any kind of co-doped elements present in the semiconductor material. This study paves the way towards the future capability of LIBS for the characterizations of semiconductor samples for their potential applications in the photonic industry.

## Figures and Tables

**Table 1:** Certified compositions (%) of Al, Ga and As present in the three AlGaAs samples.

*Species*	*Sample-1*	*Sample-2*	*Sample-3*
*Aluminium (Al)*	*3.0*	*1.00*	*0.69*
*Gallium (Ga)*	*95.0*	*96.05*	*97.32*
*Arsenide (As)*	*2.0*	*2.95*	*1.99*
∑Wt.%	*100*	*100*	*100*

**Table 2:** Major emission lines detected in the wavelength region from 200 nm to 650 nm.

*Wavelength (nm)*	*Electron Configuration Transition* *Upper Level to Lower Level*	*Upper Level Energy/E_k_* *(cm^−1^)*	*Transition Probability* *(s^−1^)*
***Gallium (Ga I)***			
*Ga I 233.82*	*6d ^2^D_5/2_→* *4p ^2^P_3/2_*	*43,580.44*	$9.75\times 10^{6}$
*Ga I 237.12*	*7s ^2^S_1/2_→* *4p ^2^P_1/2_*	*42,158.77*	$5.57\times 10^{6}$
*Ga I 241.86*	*7s ^2^S_1/2_→* *4p ^2^P_3/2_*	*42,158.77*	$1.00\times 10^{7}$
*Ga I 245.00*	*5d ^2^D_3__/2_→* *4p ^2^P_3/2_*	*40,802.86*	$2.87\times 10^{7}$
*Ga I 250.01*	*5d ^2^D_5/2_→* *4p ^2^P_3/2_*	*40,811.41*	$3.34\times 10^{7}$
Ga I 265.98 *	*6s ^2^S_1/2_→* *4p ^2^P_1/2_*	*37,584.77*	$2.44\times 10^{7}$
Ga I 271.96 *	*6s ^2^S_1/2_→* *4p ^2^P_3/2_*	*37,584.77*	$4.68\times 10^{7}$
Ga I 287.40 *	*4d ^2^D_3/2_→* *4p ^2^P_1/2_*	*34,781.66*	$4.68\times 10^{8}$
Ga I 294.36 *	*4d ^2^D_5/2_→* *4p ^2^P_3/2_*	*34,787.85*	$8.04\times 10^{8}$
Ga I 403.20 *	*5s ^2^S_1/2_→* *4p ^2^P_1/2_*	*24,788.53*	$9.70\times 10^{7}$
Ga I 417.20 *	*5s ^2^S_1/2_→* *4p ^2^P_3/2_*	*24,788.53*	$1.89\times 10^{8}$
*Ga I 639.60*	*6p ^2^P_3/2_→* *5s ^2^S_1/2_*	*40,417.62*	
*Ga I 641.30*	*6p ^2^P_1/2_→* *6p ^2^S_1/2_*	*40,376.45*	
***Arsenide (As I)***			
As I 228.81 *	*5s ^2^P_3/2_→* *4p^3^ ^2^D_5/2_*	*54,605.30*	$1.1\times 10^{9}$
As I 234.98 *	*5s ^2^P_1__/2_→* *4p^3 2^D_3/2_*	*53,135.60*	$6.8\times 10^{8}$
As I 274.49 *	*5s ^2^P_3/2_→* *4p^3 2^P_1/2_*	*54,605.30*	$1.0\times 10^{8}$
As I 278.02 *	*5s ^2^P_3/2_→* *4p^3 2^P_3/2_*	*54,605.30*	$3.1\times 10^{8}$
As I 286.04 *	*5s ^2^P_1/2_→* *4p^3 2^P_1/2_*	*53,135.60*	$1.1\times 10^{8}$
***Aluminium (Al I)***			
Al I 308.20 *	*3d ^2^D_3/2_→* *3p ^2^P_1/2_*	*32,435.45*	$2.35\times 10^{8}$
Al I 309.30 *	*3d ^2^D_5/2_→* *3p ^2^P_3/2_*	*32,436.79*	$4.37\times 10^{8}$
Al I 394.40 *	*4s ^2^S_1/2_→* *3p ^2^P_1/2_*	*25,347.75*	$9.98\times 10^{7}$
Al I 396.15 *	*4s ^2^S_1/2_→* *3p ^2^P_3/2_*	*25,347.75*	$1.97\times 10^{8}$

* Emission lines of gallium, arsenic, and aluminum used to construct the typical Boltzmann plots.

## Data Availability

The data that support the findings of this study are available from the corresponding author upon reasonable request.
